# Multiplex Lateral Flow Immunochromatographic Assay Is an Effective Method to Detect Carbapenemases in Non-Susceptible *Acinetobacter baumannii*

**DOI:** 10.3390/jcm15093232

**Published:** 2026-04-23

**Authors:** Ilay Pinto, Talya Finn, Svetlana Paikin, Jonathan Lellouche

**Affiliations:** 1 Adelson School of Medicine, Ariel University, Ariel 4070000, Israel; pintoilay@gmail.com (I.P.); tfinnfried@laniado.org.il (T.F.); 2 Infectious Diseases Unit, Sanz Medical Center, Laniado Hospital, Netanya 4244916, Israel; 3 Clinical Laboratories Department, Sanz Medical Center, Laniado Hospital, Netanya 4244916, Israel; svetlana@laniado.org.il

**Keywords:** *Acinetobacter baumannii*, carbapenem-resistance, carbapenemases, rapid diagnostic, lateral flow immunochromatographic assay

## Abstract

**Objectives:** This study evaluated the diagnostic performance of the lateral flow immunochromatographic assay (RESIST-ACINETO, Coris BioConcept) for the rapid detection of the major carbapenemases in *Acinetobacter baumannii*. **Methods:** Blood culture isolates collected between 2014 and 2024 with meropenem MIC ≥ 4 mg/L were retrieved, re-identified by MALDI-TOF MS, and susceptibility was confirmed by broth microdilution. Carbapenemase genes (*bla*_OXA-23_, *bla*_OXA-40_, *bla*_NDM_) were detected using multiplex PCR, which served as the reference standard. All isolates were tested using the RESIST ACINETO assay, and diagnostic accuracy parameters were calculated. **Results:** A total of 114 isolates were recovered and confirmed as *A. baumannii*. Among 93 carbapenem-non-susceptible isolates, 97.8% (91/93) were correctly identified by the assay. The test showed 99.1% sensitivity and 99.1% specificity, with most positive results appearing within 3–10 min. Two discrepant results were observed (one false positive, one false negative), while all meropenem-susceptible isolates tested negative. **Conclusions:** The RESIST ACINETO assay provides rapid, accurate detection of carbapenemases in *A. baumannii*, significantly reducing turnaround time compared with conventional workflows. Its performance supports integration into routine diagnostics to enhance timely resistance confirmation and infection-control interventions.

## 1. Introduction

*Acinetobacter baumannii* is an opportunistic pathogen that has emerged as a significant public health threat, particularly in hospital settings, due to its remarkable ability to acquire antibiotic resistance and its association with severe infections. Recognized as a global health concern, *A. baumannii* is associated with hospital-acquired pneumonia, bloodstream, and wound infections, particularly among critically ill patients and those who are immunocompromised [1]. Its resilience across diverse environments, capacity for biofilm formation, and ability to survive for extended periods under dry conditions facilitate its persistence and transmission in healthcare settings, thereby contributing to nosocomial outbreaks and posing significant challenges to infection control programs [2].

The rise in multidrug-resistant (MDR) clones of *A. baumannii* poses a challenge to clinicians, with few effective therapeutic options available. This resistance is attributed to the organism’s inherent genomic plasticity, allowing for mutations and the acquisition of resistance genes, which enhance the ability of *A. baumannii* to adapt to the environment [2,3]. Carbapenems, a subclass of beta-lactam antibiotics and cefiderocol, are usually considered the last line of antibiotics for treating MDR Gram-negative. The worldwide increase in the prevalence of carbapenem resistance in *A. baumannii* (CRAB) is alarming [3,4]. Since 2021, the U.S. CDC and WHO have categorized CRAB as an urgent public health threat in U.S. healthcare facilities, when it was estimated there were still 8500 cases among U.S. hospitalized patients in 2017, resulting in 700 deaths and nearly 281 million dollars in excess healthcare costs [5,6]. In parallel, the World Health Organization has prioritized CRAB for new antibiotic development due to its high morbidity and mortality rates [2,6].

The most commonly identified mechanisms resulting in CRAB include alterations of penicillin-binding proteins, decreased membrane porin channel density, increased efflux pump expression, and the production of enzymes (i.e., carbapenemases) [2]. Serine-dependent class D oxacillinase enzymes have been specifically identified among *Acinetobacter baumannii* when the more common genes detected are *bla*_OXA-23-LIKE_, *bla*_OXA-24/-40-LIKE_, and *bla*_OXA-58-LIKE_ [4]. A recent study reported that carbapenemase genes were detected in 83% of CRAB isolates tested in the U.S. [5]. A small proportion of CRAB also possessed mobile genes that encode another carbapenemase, including the *bla*_IMP_, *bla*_NDM_, and *bla*_VIM_ class B metallo-beta-lactamases, which are often found in other Gram-negative bacteria, such as Enterobacterales [4]. These carbapenemase genes can transfer horizontally among bacteria, increasing the likelihood that multidrug resistance will spread.

The currently available methods for laboratory antibiotic testing and determining minimal inhibitory concentrations (MICs) of carbapenems, generating results within 24 to 48 h following identification of a suspected colony, include the gold-standard broth microdilution [7]. However, some are labor-intensive and time-consuming [8]. It suggests the need to develop rapid diagnostic tools to shorten the time to laboratory reports, enabling targeted and appropriate treatment. Immunochromatographic tests can be implemented in a lateral flow kit, a simple, rapid, and cost-effective technique. Rapid lateral flow tests were developed and were widely implemented to detect *Legionella pneumophila* [9], toxigenic *Clostridium difficile* [10], and carbapenemase-producing Enterobacterales [11]. However, most studies evaluating immunochromatographic assays for carbapenemase detection have predominantly focused on Enterobacterales, with limited data on CRAB, highlighting the need for dedicated evaluation in this clinically relevant pathogen. A novel immunochromatographic commercial assay (RESIST ACINETO, Coris Bioconcept, Gembioux, Belgium) was recently introduced in the market. To our knowledge, it is the first rapid diagnostic assay based on lateral flow to detect the most frequently acquired carbapenemases (OXA-23, OXA-40/58, and NDM) in Acinetobacter species within 15 min [12]. The assay is based on a lateral flow immunochromatographic principle using monoclonal antibodies directed against specific carbapenemase protein epitopes, enabling direct detection of expressed enzymes at the protein level. The test targets the main clinically relevant carbapenemase families in *A. baumannii*, including OXA-23, OXA-40/58, and NDM enzymes. The device incorporates a multiplex antibody configuration, with distinct capture lines for each targeted carbapenemase, allowing simultaneous detection and differentiation. Briefly, a bacterial colony is suspended in an extraction buffer and applied to the test cassette; the lysate migrates along the membrane by capillary action, where carbapenemase antigens, if present, bind to labeled antibodies and are captures on specific test lines, producing a visible signal within minutes. Here, we aim to evaluate the laboratory performance of the RESIST ACINETO assay for the confirmation of the presence of carbapenemases in *A. baumannii*.

## 2. Materials and Methods

**Sample design and composition.** A list was made of all blood cultures that tested positive for non-susceptible *A. baumannii* between 2014 and 2024. The isolates were selected following initial identification as *A. baumannii* (VITEK MS, Biomerieux, Mercy L’Etoile, France) or *A. baumannii* complex (VITEK 2, Biomerieux, Mercy L’Etoile, France) with a minimum inhibitory concentration (MIC MEM) for meropenem of ≥4 mg/L. Frozen isolates were located and identified for future investigations. Isolates not found in the library were excluded from the laboratory performance evaluation.

ISO 20776-2:2022 guidelines were partially used to determine the sample size, which should include *A. baumannii* isolates when a proportion of 75% isolates should be non-susceptible (MIC MEM = 4 mg/L) or resistant (MIC MEM ≥ 8 mg/L) and 25% isolates should be susceptible (MIC MEM ≤ 2 mg/L) [13]. The susceptible isolates were chosen randomly using initial MIC MEM from our library and included in the study.

**Phenotype confirmation.** All isolates were stored at −80 °C in tryptic soy broth (Hy Laboratories, Rehovot, Israel) with 15% glycerol. Before testing, each isolate was thawed and transferred twice onto Mueller–Hinton agar (Hy Laboratories, Rehovot, Israel). The bacterial identification was confirmed at the species level by Maldi-ToF (Vitek MS, bioMérieux, Marcy-l’Étoile, France). Meropenem susceptibility was confirmed by retesting using broth microdilution (BMD) [14]. The susceptibility testing for each isolate was performed twice under the same conditions on the same day. Plates were read for each isolate, and MIC were determined after incubation at 35–37 °C for 16–20 h. Susceptibilities were interpreted using the CLSI clinical breakpoints [14]. *E. coli* ATCC 25922 was used as the control strain [14]. In the case of discrepancy between the initial MIC and BMD, the isolate was retested using BMD to determine the MIC.

**Bacterial typing and detection of carbapenemase genes.** *A. baumannii* typing was performed by polymerase chain reaction (PCR) for *bla*_OXA-51_ and *gyrB* genes. The sequencing of the *bla*_OXA-51-like_ gene was used to assign isolates to international clonal lineages (IC) [15]. The presence of *bla*_OXA-23_, *bla*_OXA-40_, and *bla*_NDM_ genes was confirmed by a multiplex PCR described previously [16]. PCR was considered the gold-standard method for determining the laboratory performance of the evaluated assay.

**Figure 1:**
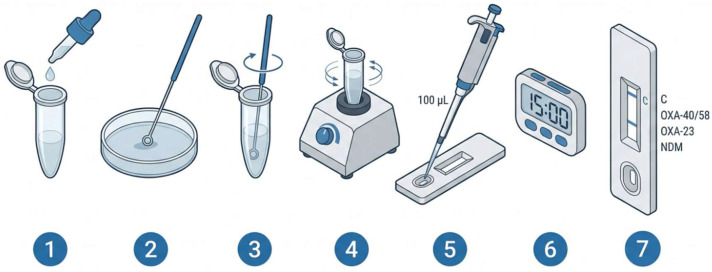
**Schematic representation of sample preparation and lateral flow immunochromatographic assay procedure**. A bacterial colony is collected from culture and suspended in an extraction buffer to release carbapenemase enzymes (1–2). The prepared lysate (3–4) is then applied to the sample well of the lateral flow cassette (5). Through capillary migration, the sample interacts with labeled antibodies and flows along the membrane, where specific carbapenemase antigens (e.g., OXA-23-like, OXA-40/58-like, and NDM) are captured by immobilized antibodies at distinct test lines. The appearance of visible bands indicates the presence of the corresponding carbapenemase, while a control line confirms the validity of the test. Results are obtained within 15 min (6–7).

**Immunochromatographic assay evaluation.** The isolates were tested in accordance with the RESIST ACINETO assay manufacturer’s instructions (Figure 1) [17]. Bacterial colonies were diluted manually in a buffer lysis. Following homogenization, 100 µL of the solution was transferred to the sample well. The results were read after 15 min and were interpreted as follows: (i) negative test result—a reddish-purple line appeared across the central reading window at the “Control” line position and no other band was present; (ii) positive test result—in addition to a reddish-purple line at the control line, a visible reddish-purple line appeared at one of the test lines positions (“NDM” or “OXA-23” or “OXA-40/58”) on the cassette; and (iii) invalid test result—the absence of a control line indicated a failure in the test procedure, and, in this case, the test was repeated with a new test device. Intensity and time to appearance of a readable positive test line were recorded. The interpretation of the immunochromatographic assay was performed by an operator blinded to the initial PCR results, in order to exclude potential reading bias or influence from prior knowledge of the molecular findings.

In cases of discrepancy between the initial PCR result and the immunochromatographic assay, the immunochromatographic assay test was repeated. If the discrepancy persisted, the PCR assay was subsequently repeated. Final results were interpreted based on the PCR result, which was considered the reference standard. Sensitivity and specificity, as well as positive and negative predictive values, were calculated.

**Ethics approval.** The study was approved by the Institutional Review Board (IRB), LND-0081-24.

## 3. Results

A total of 140 positive blood cultures with *A. baumannii* or *A. baumannii* complex were identified from the electronic database. Among these, 114 isolates (69.9%, 114/140) were successfully recovered from frozen stocks and included in the study. All isolates (n = 114) were confirmed as *A. baumannii* by Maldi-ToF and with the presence of both OXA-51 and gyrB genes. Meropenem susceptibility testing showed that 21 isolates (18.4%, 21/114) were susceptible, four (3.5%, 4/114) were intermediate, and 89 (78.1%, 89/114) were resistant. Details of the *A. baumannii* isolates are shown in Table 1.

Among the susceptible isolates, minimum inhibitory concentrations (MICs) ranged from 0.125 to 1 mg/L. Four intermediate isolates (3.5%, 4/114) exhibited an MIC of 4 mg/L. Resistant isolates (n = 89) displayed MICs ranging from 8 to ≥64 mg/L: 8 mg/L, 11 isolates (12.4%, 11/89); 16 mg/L, 23 isolates (25.8%, 23/89); 32 mg/L, 27 isolates (30.3%, 27/89); and ≥64 mg/L, 28 isolates (31.5%, 28/89) (Table 1).

Carbapenemase profiling revealed that 58 isolates (62.4%, 58/93) carried a single carbapenemase gene: 28 isolates (30.1%, 28/93) harbored OXA-23, 28 isolates (30.1%, 28/93) harbored OXA-40, and two isolates (2.2%, 2/93) harbored NDM. In addition, 30 isolates (32.3%, 30/93) were positive for both OXA-23 and OXA-40, while four isolates (4.3%, 4/93) carried both NDM and OXA-23. Further analyses of the OXA allelic variants showed that among the isolates with only OXA-23 (n = 28), 19 (67.9%, 19/28) were OXA-66, eight (28.6%, 8/28) were OXA-71, and one (3.6%, 1/28) belonged to another OXA type. Among isolates with only OXA-40 (n = 28), 23 (82.1%, 23/28) were OXA-66, two (7.1%, 2/28) were OXA-71, and three (10.7%, 3/28) belonged to other types. Of the isolates carrying both OXA-23 and OXA-40 (n = 30), 17 (56.7%, 17/30) were OXA-66, six (20.0%, 6/30) were OXA-71, and seven (23.3%, 7/30) were other types. Among the isolates where NDM was detected (n = 6), the two isolates harboring only NDM were classified as other OXA types (33.3%, 2/6), while among the four isolates carrying NDM together with another carbapenemase, two were OXA-71 (33.3%, 2/6), one was OXA-66 (16.7%, 1/6), and one was another OXA type (16.7%, 1/6) (Table 1).

**Figure 2:**
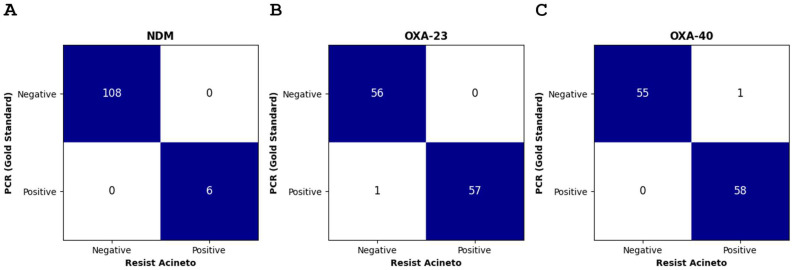
**Comparison of lateral flow immunochromatographic assay with PCR.** Comparison of lateral flow immunochromatographic assay results (n = 114) for (**A**) NDM, (**B**) OXA-23, and (**C**) OXA-40 carbapenemases. Blue squares indicate concordant results between the lateral flow assay and PCR (gold standard), while white squares represent isolates showing a discrepancy between the two methods.

The RESIST ACINETO assay accurately detected carbapenemase production in 91 (97.8%, 91/93) CRAB isolates expressing a carbapenemase that the assay is designed to detect (NDM, OXA-23, and OXA-40), including isolates that were double carbapenemase producers (Figure 2). Although results were read after 15 min, as per the manufacturer’s instructions, in most cases a positive result was obtained after 3:00 min for OXA-23, 4:10 min for OXA-40, and 10:25 min for NDM (Table 2). Two discrepancies were observed and confirmed when an isolate tested positive for OXA-23 by PCR was not detected by the RESIST ACINETO assay (false negative) and an isolate tested positive for OXA-40 by PCR the RESIST ACINETO assay but negative by PCR (false positive) (Figure 2). All 21 CRAB isolates susceptible to meropenem were correctly tested negative. In our sample, the RESIST ACINETO assay had 99.1% sensitivity (95% CI: 95.2–100.0%) and 99.1% specificity (95% CI: 95.2–100.0%).

## 4. Discussion

In this study, we demonstrate that the evaluated immunochromatographic assay exhibits high diagnostic performance for the reliable detection of the major carbapenemases in CRAB. The assay successfully identified carbapenemase-producing isolates with high confidence, accurately reflecting resistance to carbapenems and supporting its utility in the diagnostic workflow. In addition, the simplicity and rapidity of the assay supports its seamless integration into routine laboratory workflows with the minimal additional workload or technical requirements.

Our findings are consistent with results reported in previous studies evaluating this assay, confirming both the high sensitivity and specificity of the assay in detecting carbapenemases in *A. baumannii* and *A. baumannii* complexes [12,18,19]. This strong specificity is attributable to the high-affinity antibody-based design of the lateral flow immunoassay, which detects carbapenemase protein epitopes directly. Detection at the protein level is less sensitive to genetic variations, whereas molecular PCR-based assays rely on gene-targeting, making them more susceptible to genetic drift, mutations, or novel gene variants. Given the well-described genomic plasticity of *Acinetobacter baumannii*, protein-based approaches may therefore provide improved reliability and mitigate potential false-negative results caused by divergent or uncharacterized resistance determinants. Additionally, immunochromatographic assay-positive/PCR-negative discrepancies may also be explained by cross-reactivity or non-specific antibody binding, as well as differences in detection levels, since protein-based detection may capture expressed enzymes even when genetic targets are not amplified by PCR. Conversely, immunochromatographic assay-negative/PCR-positive results may occur in cases of low-level carbapenemase expression, reduced protein production, or antigen concentrations below the detection threshold of the immunochromatographic assay despite the presence of the corresponding gene.

Traditionally, the laboratory diagnostic methodology for confirming CRAB has required multiple sequential steps: utilization of selective and chromogenic culture media, identification of suspected colonies, and phenotypic confirmation of reduced susceptibility to carbapenems (e.g., imipenem or meropenem). This workflow inherently delays final confirmation, as phenotypic verification requires an additional incubation period (~18 h) before a report can be issued. In contrast, the implementation of the lateral flow immunoassay enables near-immediate carbapenemase detection directly from the suspected colony, producing validated results within minutes. This approach eliminates the need to wait for phenotypic confirmation of carbapenem resistance by standard culture methods, thereby significantly shortening the time to report. This significant reduction in diagnostic turnaround time has practical clinical implications. Rapid availability of results allows earlier implementation of targeted infection control measures and supports timely optimization of antimicrobial therapy in patients with confirmed CRAB infections. This improvement in turnaround time is especially relevant in clinical settings with high CRAB prevalence, where rapid confirmation supports earlier implementation of infection-control strategies, reducing transmission risk and improving containment effectiveness. From an economic perspective, the lateral flow assay also represents a cost-effective alternative, with an estimated cost of approximately 10 euros per test compared to around 20 euros for PCR-based methods.

In this context, our study is based on a relatively large collection of isolates recovered from blood cultures over an extended period, reflecting the local epidemiology of CRAB. This setting is representative of Israel, a region known to be endemic for CRAB, with epidemiological characteristics comparable to those reported in several European countries such as Italy and Greece, thereby supporting the clinical relevance and broader applicability of our findings.

Despite the strengths of this study, several limitations should be taken into consideration. Although the analysis demonstrates the potential clinical value of rapid carbapenemase detection, our study did not evaluate the real implementation of this assay in our setting. As a result, the inferred clinical benefits, such as faster cohorting, reduced unnecessary antimicrobial exposure, or shortened hospitalization, remain theoretical rather than directly observed in practice. Future prospective clinical studies employing lateral flow immunochromatographic assay testing in routine diagnostics will be required to quantify the real benefits. Additionally, our sample reflects CRAB epidemiology specific to the Israeli clinical setting, which may limit these findings to regions with different epidemiology and resistance profiles. Furthermore, all isolates included in this study were obtained from blood cultures and recovered from frozen stocks, which may introduce bias in the evaluation of test performance compared to fresh, prospectively collected clinical samples. Additionally, not all mechanisms underlying carbapenem resistance are covered by the evaluated assay. As a targeted test, it is restricted to the detection of selected carbapenemase families (NDM, OXA-23-like, and OXA-40/48-like) and therefore may not identify resistance mediated by other carbapenemases or non-enzymatic mechanisms. Finally, relevant operational aspects, including cost-effectiveness and laboratory integration, were not evaluated in this study. These parameters represent essential considerations for implementation and should be assessed to support evidence-based adoption of rapid diagnostics at scale.

## Figures and Tables

**Table 1:** Details of *A. baumannii* isolates characterized by PCR.

Clone	No. of Isolates n (%)	OXA-23 n (%)	OXA-40 n (%)	NDM n (%)	Combination (n, %)	MEM MIC Range (mg/L)
**Non-Susceptible AB**	93 (81.6)	58 (50.8)	58 (50.8)	6 (5.3)	34 (29.8)	4–64
OXA-66 (ST2)	62 (54.4)	36 (31.6)	40 (35.1)	1 (0.9)	18 (15.8)	4–64
OXA-71 (ST3)	17 (14.9)	14 (12.3)	8 (7.0)	2 (1.8)	8 (7.0)	4–64
Others	14 (12.3)	8 (7.0)	10 (8.8)	3 (2.6)	8 (7.0)	16–64
**Susceptible AB**	21 (18.4)	-	-	-	-	0.125–1
**Total**	**114 (100.0)**	**58 (50.8)**	**58 (50.8)**	**6 (5.2)**	**34 (29.8)**	

Abbreviation: AB—*A. baumannii*; MEM—meropenem; MIC—minimum inhibitory concentration, ST—sequence types 2/3 corresponding to the international clone II and III.

**Table 2:** Time to visible positive line for carbapenemases using the lateral flow immunochromatographic assay.

Time to Detection	Min (Minutes)	Max (Minutes)	Median (Minutes)
NDM	5:20	12:00	10:25
OXA-23	1:00	8:21	3:00
OXA-40	1:00	14:00	4:10
Sample	1:00	14:00	3:20

Observed minimum, maximum, and median times required for the lateral flow immunochromatographic assay to display a detectable positive line for NDM, OXA-23, and OXA-40 carbapenemases. Times represent minutes from initial sample application to visual interpretation of a positive result. Abbreviations: Min—minimum, Max—maximum.

## Data Availability

The original contributions presented in this study are included in the article. Further inquiries can be directed to the corresponding author.
